# Do Bankart lesions heal better in shoulders immobilized in external rotation? A randomized single-blind study of 55 patients examined with MRI

**DOI:** 10.3109/17453670903278266

**Published:** 2009-10-01

**Authors:** Sigurd Liavaag, Morten Georg Stiris, Elisabeth Stokke Lindland, Martine Enger, Svein Svenningsen, Jens Ivar Brox

**Affiliations:** ^1^Department of Orthopedic Surgery, Sørlandet HospitalArendalNorway; ^2^Department of Diagnostic Radiology, Oslo University HospitalOsloNorway; ^3^Department of Radiology, Sørlandet HospitalArendalNorway; ^4^Orthopedic Center, Skadelegevakten, Oslo University Hospital and Medical School, University of OsloOsloNorway; ^5^Department of Orthopedic Surgery, RikshospitaletOsloNorway

## Abstract

**Background and purpose** Immobilization in external rotation (ER) for shoulder dislocation has been reported to improve the coaptation of Bankart lesions to the glenoid. We compared the position of the labrum in patients treated with immobilization in ER or internal rotation (IR). A secondary aim was to evaluate the rate of Bankart lesions.

**Patients and methods** 55 patients with primary anterior shoulder dislocation, aged between 16 and 40 years, were randomized to immobilization in ER or IR. Computer tomography (CT) and magnetic resonance imaging (MRI) were performed shortly after the injury. After the immobilization, MRI arthrography was performed. We evaluated the rate of Bankart lesions and measured the separation and displacement of the labrum as well as the length of the detached part of the capsule on the glenoid neck.

**Results** Immobilization in ER reduced the number of Bankart lesions (OR = 3.8, 95% CI: 1.1 –13; p = 0.04). Separation decreased to a larger extent in the ER group than in the IR group (mean difference 0.6 mm, 95% CI: 0.1 – 1.1, p = 0.03). Displacement of the labrum and the detached part of the capsule showed no significant differences between the groups.

**Interpretation** Immobilization in ER results in improved coaptation of the labrum after primary traumatic shoulder dislocation.

## Introduction

Recurrent dislocations are common after traumatic anterior glenohumeral dislocation and age at the time of primary dislocation is reported to be the most important risk factor (Henry and Genung 1982, Hovelius et al. 1996, Kralinger et al. 2002). Reduction followed by a period of 2–3 weeks with the arm in a sling is still an accepted treatment of primary dislocation, also in young patients. The literature shows no concensus regarding the benefit of traditional immobilization (Kiviluoto et al. 1980, Henry and Genung 1982, Hovelius et al. 1996, Kralinger et al. 2002, Itoi et al. 2007). Based on a cadaveric study, Itoi et al. (2000) introduced the term coaptation zone, in which the edges of a simulated Bankart lesion were kept approximated without the surrounding muscles. These authors anticipated that choosing a position of immobilization (within the so-called coaptation zone) that increases tension in the anterior soft tissue (such as adduction and external rotation) may be better than immobilizing the shoulder in the conventional position. In another study (Itoi et al. 2001), they found that external rotation increases the amount of coaptation compared to internal rotation. They postulated that in external rotation the subscapularis muscle becomes tight, which may prohibit the development of a hematoma and promote better coaptation. In an earlier prospective randomized study (Wintzell et al. 1999), it was reported that removal of the hematoma with arthroscopic lavage reduced the recurrence rate following primary anterior shoulder dislocation. Limpisvasti et al. (2008) questioned the importance of coaptation. In their cadaveric study, external rotation did not have any effect on the contact pressure between the subscapularis muscle and the labrum. Contrary to this, Seybold et al. (2009) found that immobilization in external rotation after first-time dislocation improved the position of the labroligamentous lesion on the glenoid rim. A recent randomized controlled study by Itoi et al. (2007) has shown that immobilization with the arm in external rotation after shoulder dislocation reduces the risk of recurrence compared with conventional immobilization in internal rotation. Itoi's cross-sectional MRI study (Itoi et al. 2001) included patients with initial dislocation and patients with recurrent dislocation. The patients were only evaluated once after the dislocation, and the study was not designed to test whether the effect of the position of the labrum was permanent or temporary.

In the present prospective study, the aim was to test whether 3 weeks of immobilization in external rotation approximates the Bankart lesion to the glenoid neck differently than immobilization in internal rotation. To evaluate changes in the outcome parameters, imaging was done both shortly after the injury and after the immobilization was finished.

## Patients and methods

**Figure 1.**
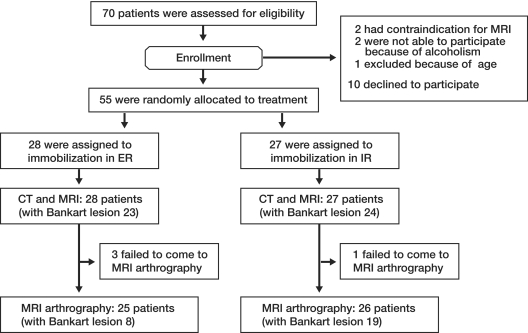
Flow chart of the prospective randomized multicenter study. Immobilization in internal versus external rotation after primary anterior traumatic shoulder dislocation. Age group 16–24 years: n = 82. Age group 25–40 years: n = 106. Total: 188 patients included. 67 patients were diagnosed and treated at Oslo casualty ward (Oslo University Hospital, Ullevaal). 3 patients were diagnosed and treated at Akershus University Hospital. Patients from these 2 hospitals were also recruited to the CT/MRI/MRI arthrography study.

In February 2005, we started a prospective randomized multicenter study in Norway to compare the recurrence rate of shoulder dislocation in 2 groups of patients. Patients with initial traumatic anterior shoulder dislocation were randomized to either immobilization with the arm in external rotation (ER) or immobilization with the arm in internal rotation (IR). The inclusion of patients was finished in February 2008. 188 patients from 13 hospitals were included in the study. At 2 centers (Oslo University Hospital, Ullevaal, and Akershus University Hospital) the patients were also asked to participate in the present study. This included examination of the shoulders by CT and MRI at the beginning of the treatment and by MRI arthrography (MR-a) after the treatment was finished. Of the 70 patients who were diagnosed and treated at these 2 hospitals, 55 fulfilled the inclusion criteria, were willing to participate in the present study, and gave their informed consent (Figure 1).

The inclusion criteria were as follows: patients aged 16–40 years with primary traumatic anterior glenohumeral dislocation successfully reduced and documented by conventional radiographic examination before and after reduction.

The exclusion criteria were: (1) fracture of the glenoid with a large bony defect of the glenoid rim (including more than 20% of the length of the glenoid rim) (Burkhart and De Beer 2000), or a bony glenoid defect involving more than one-third of the diameter of the glenoid fossa at the same level; (2) fracture of the greater tubercle of the humerus, with malalignment after repositioning; (3) nerve damage related to dislocation or reduction (plexus damage or damage of axillary nerve); and/or (4) the patient was not willing to or able to go through the investigation.

The mean age of the patients included was 27 years (SD 7) (Table 1). There were 14 patients older than 25 years in each group. Allocation to treatment in ER or IR was conducted directly after reduction of the dislocated shoulder. Block randomization was conducted at each hospital that recruited patients. We did not stratify for sex. Concealed randomization was performed according to the method described by Altman (1991) and sealed envelopes were used.

**Table 1.** Demographic characteristics of the patients

Variable	All patients	ER group	IR group
Sex			
Men	41	18	23
Women	14	10	4
Mean age (SD)			
All	27 (7)	27 (8)	27 (6)
Men	26 (7)	25 (7)	28 (6)
Women	29 (7)	30 (8)	26 (5)
Mean days (SD) from injury			
MRI/CT	7 (3)	7 (3)	7 (3)
MRI arthrography (n = 51)	54 (73)	49 (67)	59 (79)

For reduction of the dislocated shoulder, a method called the Urnes method was used in 49 cases. This is a technique with vertical traction and external rotation; it has been used for many years at Oslo University Hospital, Ullevaal, and is very similar to the technique described later as the Spaso technique (Yuen et al. 2001). The method of Kocher was used in 3 cases, and in another 3 cases the Stimson's hanging arm method (Stimson 1900, Thakur and Narayan 1990, Cunningham 2005, Chitgopkar and Khan 2005) was used. In a cadaveric study, Miller et al. (2004) found that the contact force between the Bankart lesion and the glenoid is at its maximum with the shoulder in 45˚ of external rotation. Itoi et al. (2007) reported discomfort in patients applying too much external rotation, and used 10˚ of external rotation in his prospective randomized study. We had tested our immobilizer in a small pilot study and experienced that the patients tolerated immobilization in 15˚ external rotation very well. All patients in the ER group used a prefabricated shoulder immobilizer (Don Joy Ultrasling ER, 15˚ version). All 3 available sizes (small, medium, and large) were stored in the hospital in a left and right version. To control the position, a line at the top of the immobilizer should be parallel with the frontal plane when the arm is correctly placed. The hospital personnel were trained to instruct patients to use the immobilizer. All patients in the IR group used an ordinary collar and cuff device. After they had finished the immobilization, all patients were asked in a standard questionnaire if they had any problems with the use of the immobilizer or the collar and cuff. It was not possible to provide MRI and CT immediately, but these examinations were conducted shortly after the treatment was started. The median length of time between the dislocation and the CT and MRI was 7 (2–14) days. When MRI is undertaken during the first 2 weeks after the dislocation, the blood and effusion in the joint cavity will act as a contrast fluid. After the immobilization period, patients were investigated with MRI arthrography. All 55 patients had CT and MRI. For different reasons, 4 patients did not have MRI arthrography (Figure 1).

The study was approved by our institutional study board, the Regional Medical Ethics Committee, and the Norwegian Social Sciences Data Services. The study was also reported to Clinical Trials.gov as part of an ongoing randomized multicenter study (Clinical Trials.gov identifier NCT00202735).

### Imaging

Imaging was conducted at the Department of Radiology, Aker University Hospital. All images were routinely reported and a standard questionnaire was used to register CT, MRI, and MRI arthrography findings. One radiologist (MGS), who was blinded as to treatment, filled in the questionnaire. He reported whether the Bankart lesion was present or not. Later, all measurements of separation and displacement of the labrum from the glenoid neck and the length of the detached part of the capsule on the glenoid neck were performed by another radiologist (ESL), who was also blinded regarding treatment. For CT examination, a multidetector scanner (Somatom Sensation 64; Siemens AG, Erlangen, Germany) was used. The patients lay supine on the CT table. An axial volume uptake (64 mm × 0.6 mm) through the acromioclavicular (AC) and glenohumeral joints was performed. Then an oblique coronal and an oblique sagittal projection were reconstructed directly from the raw data.

The MRI and MRI arthrography examinations were performed on a 1.5-tesla scanner (Siemens Symphony; Siemens AG, Erlangen, Germany). For both examinations we used a small flex coil, which was placed at 45 degrees obliquely to the long axis of the body. The following sequences were obtained in the MR evaluation: oblique coronal PD/T2-weighted sequence (FoV 200 × 150, matrix 192 × 192, TR 3,070, TE 13, TE 79) and STIR (T1 inversion recovery, FoV 200 × 159.4, matrix 157 × 256, TR 5,000, TE 29), axial PD fs (fat saturation) (FoV 175 × 175, matrix 248 × 266, TR 3000, TE 13), and sagittal oblique T2 (FoV 190 × 190, matrix 192 × 192, TR 3500, TE 88). A 4-mm slice thickness with 0.8-mm gap and an acquisition of 1 were used in all sequences.

The MRI arthrography examination was performed fluoroscopically under sterile conditions. The patient lay supine on the fluorotable. Under fluoroscopic guidance, a 19-G needle was introduced into the joint. 5 mL of Omnipaque 300 (General Electric) and 10 mL of diluted (0.02 mmol/L) Omniscan (General Electric) were injected. An arthrogram was then obtained, using the following sequences: oblique coronal PD/T2 (FoV 200 × 150, matrix 192 × 256, TR 3,000, TE 15, TE 93) and T1 fs (FoV 175 × 131.3, matrix 146 × 256, TR 490, TE 12), axial T1 fs (FoV 160 × 160, matrix 256 × 256, TR 490, TE 12), oblique sagittal T1 (FoV 160 × 1,660, matrix 256 × 256, TR 558, TE 12), and ABER (abduction external rotation) T1 (FoV 160 × 160, matrix 256 × 256, TR 551, TE 12). The slice thickness was 4 mm with a gap of 0.8 mm, with an acquisition of 2. The MRI and MRI arthrography examinations were sent to the PACS archive for evaluation.

To achieve approximately the same degree of rotation in all patients on CT, MRI, and MRI arthrography, the patients kept the arm along the side of the trunk with the thumb facing upwards.

### Measurements

**Figure 2.**
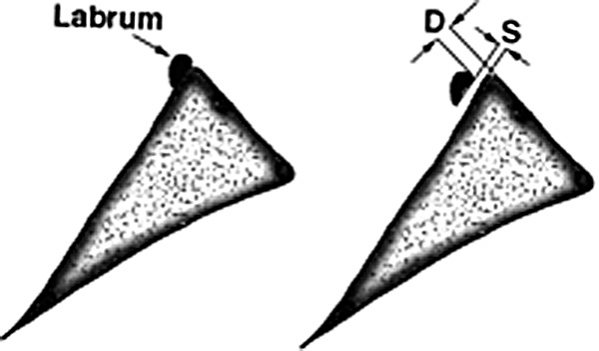
The definition of ‘separation’, ‘displacement’ and ‘detached length’ was done according to the method of Eiji Itoi. Figures are reproduced (by permission of the author and the Journal of Bone and Joint Surgery) from the original article of Itoi in JBJS (AM) Number 5. May 2001. Separation (S) was defined as the distance (in mm) between the inner margin of the labrum and the anterior aspect of the glenoid neck. Displacement (D) was defined as the distance (in mm) between the tip of the labrum and the tip of the glenoid rim. The value was positive when the labrum was displaced medial to the rim of the glenoid.

**Figure 3.**
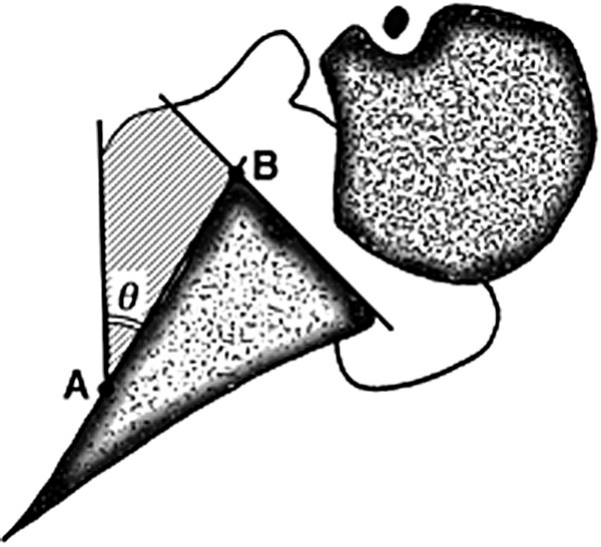
Measurements of the capsule: The detached length (A to B) is the length between the anterior glenoid rim and the anterior capsular attachment (in mm). The figure is reproduced (by permission of the author and the Journal of Bone and Joint Surgery) from the original article of Itoi in JBJS (AM) Number 5. May 2001. In our study the detached area (lined area) and the opening angle θ were not measured.

Measurements of the separation and displacement of the labrum and also measurements of the detached length of the capsule were conducted according to the method of Itoi et al. (2001) (Figures 2 and 3). The data were normalized by dividing the observed values by the ratio of the measured diameter of the humeral head to the mean diameter of the humeral head. We assumed that the detached area and the opening angle are influenced by the degree of humeral rotation and the amount of intraarticular fluid. We found it difficult to measure the exact opening angle between the anterior aspect of the glenoid neck and a line tangential to the capsule at the glenoid insertion, and did not measure these parameters as Itoi et al. had done in their original study (Figure 3).

All data were measured twice and the mean value of the 2 measurements was used. For measurements of separation, displacement, and detached length the intraclass correlation coefficients ranged from 0.91 (95% CI: 0.85–0.95) to 0.99 (95% CI: 0.98–0.99). We estimated measurement error by calculating the repeatability coefficient, multiplying the within-subject standard deviation by 1.96√2 (Bland and Altman 1999). 2 readings by the same method will be within the repeatability coefficient for 95% of subjects. The repeatability coefficients for MRI/MR-a were as follows: separation 1.1/1.0 mm; displacement 1.5/1.3 mm; detachment 4.3/2.4 mm.

The Bankart lesion was defined as an avulsion of the anterior capsulolabral complex inferior to the equator of the glenoid. Anterior periostal sleeve avulsion (ALPSA) was also registered as being present or not.

We used the definitions of Itoi et al. (2001) to describe the position of the labrum relative to the glenoid. Separation (S) is the distance in mm between the inner margin of the labrum and the anterior aspect of the glenoid neck. Displacement (D) is the distance in mm between the tip of the labrum and the tip of the glenoid rim. The value was defined as positive when the labrum was displaced medial to the rim of the glenoid and negative when the labrum was displaced laterally, towards the humeral head (Figure 2). The detached length of the capsule was defined as the length in mm between the anterior glenoid rim and the anterior capsule attachment (Figure 3).

It was not possible to measure distances in all patients. The reason given by the radiologists was a poorly defined labrum or capsule attachment, and in one patient there was failure of fat suppression in the STIR sequence.

### Statistics

The estimated sample size was based on results from the MRI study of Itoi et al. (2001). We also supposed a spontaneous healing rate, regardless of treatment, of 10%. We designed the study to detect a 40% difference between the groups on MRI arthrography. With α = 0.05 and β = 0.2, we estimated that 20 patients would be required in each group.

Adjustments were made for baseline values, age, and sex using a general linear model or logistic regression. All data were checked for the shape of the distribution. The analyses were performed according to the principle of intention to treat.

## Results

All patients used the immobilizer more than 16 h every day and night for 3 weeks. After these 3 weeks 13 patients in the ER group and 10 patients in the IR group reported problems with the use of the immobilizer.

The median length of time between dislocation and MRI arthrography was 31 (21–385) days. 1 male patient in the ER group had the MRI arthrography 11 months after the injury and 1 male patient in the IR group had it 7 months after the injury. None of these 2 patients had new traumas or redislocations, but their Bankart lesions were not healed. One female patient in the IR group had the MRI arthrography after 1 year and her Bankart lesion was healed.

### Labral lesions, healing of the Bankart lesion, labral coaptation, and detachment of the capsule

47 of the 55 patients had a Bankart lesion on the initial MRI, and 27 patients still had the Bankart lesion when examined with MRI arthrography. There was a difference (p = 0.04) between the groups in favor of ER, with an adjusted OR of 3.8 (95% CI: 1.1–13.3) (Table 2). 3 patients in the IR group and 5 in the ER group had no Bankart lesion.

**Table 2.** Bankart lesions detected in MRI and in MRI arthrography

Bankart lesion	ER group	IR group	Adjusted OR (95% CI) ^a^
MRI (male)	23 (16)	24 (21)	
MRI arthrography (male)	8 (6)	19 (18)	3.8 (1.1–13) **^b^**
^a^ Adjustments were made for baseline values, sex, and age.				^b^ In the intention-to-treat analysis, we used a conservative approach and considered that 4 patients who did not attend follow-up still had a Bankart lesion (p = 0.04).			

All Bankart lesions on MRI arthrography were also visible on the initial MRI. In 7 patients, the Bankart lesion was transformed from a Bankart lesion on MRI to an anterior periostal sleeve avulsion (ALPSA) lesion on MRI arthrography. We did not find any patients with an ALPSA lesion on the initial MRI, and there was no statistically significant difference between the groups concerning the rate of ALPSA lesions.

In 9 patients it was not possible to conduct all measurements of separation, displacement, and detached capsule length. Four patients failed to come to MRI arthrography. In 4 other patients, it was not possible to measure separation and displacement on MRI and on MRI arthrography because the labrum was not detectable or poorly defined, and in 1 patient it was not possible to measure the detached length.

The mean adjusted difference in separation on MRI arthrography between the groups was 0.6 mm (95% CI: 0.1–1.1, p = 0.03) (Table 3). We did not find any differences between groups in displacement and in the detached length of the capsule (Figure 2 and Table 3).

**Table 3.** Normalized measurements (mm) of separation and displacement of the labrum and the detached length of the capsule

	ER mean (SD)	IR mean (SD)	Adjusted mean diff. (95% CI), p-value ^a^
Separation			
MRI	0.8 (1.1)	0.8 (0.8)	
MRI arthrography	0.4 (0.8)	1.0 (1.0)	0.6 (0.1–1.1), 0.03
Displacement			
MRI	0.5 (2.1)	1.5 (2.3)	
MRI arthrography	0.6 (1.5)	0.9 (1.4)	0.1 (-0.8–0.7), 0.8
Detached length			
MRI	13.6 (6.4)	14.9 (5.9)	
MRI arthrography	9.8 (5.0)	10.0 (5.5)	0.3 (-2.7–3.4), 0.8
^a^ Adjustments were made for baseline values, sex, and age.			

### Fractures and Bankart lesions

6 patients had a fracture of the greater tuberosity, 2 in the ER group and 4 in the IR group. In the IR group 3 patients had a Bankart lesion on MRI; none of these were healed on MRI arthrography. Only 1 of the patients in the ER group had a Bankart lesion and this was healed on MRI arthrography. 4 of the 6 fractures were described as comminute without displacement of the fragments; 2 were described only as undisplaced fractures of the greater tuberosity.

## Discussion

The most important findings in the present study were the reduced number of Bankart lesions and the reduced separation in the ER group. These findings are in agreement with results previously published by Itoi et al. (2001). The difference in separation between groups was statistically significant, but smaller than the measurement error. Thus, the clinical significance is difficult to interpret. In contrast to Itoi et al., we found no differences in displacement of the labrum and capsular coaptation (detached length).The major advantage of our study is the randomized design and the blinded follow-up evaluations in order to reduce selection and observer bias. The experience level of radiologists can affect reproducibility and accuracy (Waldt et al. 2005, van Grinsven et al. 2007). All our images were described by one experienced musculoskeletal radiologist (MGS), who filled in a standard questionnaire. Another radiologist (ESL) performed the measurements. Both radiologists were blinded to the treatment given.

The major limitation of our study is the time from the start of treatment to the baseline MRI evaluation. Comparing our results with those of Itoi et al. (2001), it is possible that more adaptations may have occurred in the ER group in the initial period. Thus, our observations may underestimate the real difference, and may also contribute to the observed minor differences for displacement on MRI arthrography.

In the study by Itoi et al. (2001), the mean length of time between the last dislocation and the MRI was 4 days for the patients who had a primary dislocation, but it was 29 days for the patients who had a recurrent dislocation. However, Itoi did not measure changes over time. Another limitation both of our study and the study by Itoi et al. is that separation, displacement, and detached length could not be measured in all patients, and that MRI arthrography could not be performed successfully on 4 patients in our study.

In contrast to Itoi et al. (2001) and Seybold et al. (2009), we performed measurements in the anticipated neutral position and evaluated changes in the position of the labrum over time. We controlled the degree of rotation by letting the supine patient hold the arm along the side of the trunk with the thumb facing upwards. We did not measure the exact angle. The ABER position (abducted and externally rotated) was used only to detect if a Bankart lesion was present or not. According to Itoi et al. (2001), the extent of rotation plays a role in the distance of separation and displacement. By applying a randomized study design, measurement error attributed to position is likely to be evenly distributed between groups. However, we cannot exclude that slight differences in arm positioning may have influenced our results.

Itoi et al. (2001) included patients with primary and recurrent dislocation, but as with the study by Seybold et al. (2009) we only included primary dislocations. Seybold et al. classified the labroligamentous lesions as Bankart lesions, Perthes lesions, and non- classifiable lesions. In our study and in the study by Itoi, there was no differentiation between Bankart lesions and Perthes lesions.

In the present study the patients in the ER group were immobilized in 15˚ of external rotation. Although many of the patients found the immobilizer impractical to use, the compliance was good in both groups.

Previous studies have found age to be the most important factor in determining the risk of recurrent instability (Henry and Genung 1982, Hovelius et al. 1996, Kralinger et al. 2002, Robinson et al. 2006). Diverging results have been reported for gender (Hovelius et al. 1996, Robinson et al. 2006). Our results were adjusted for both age and gender.

The differences between our results and those of Itoi et al. may be attributed to study design. The clinical importance of our findings will be evaluated in a larger ongoing randomized study with 2 years of follow-up.
